# Development of a new model for rotator cuff pathology: the rabbit subscapularis muscle

**DOI:** 10.1080/17453670902807425

**Published:** 2009-02-01

**Authors:** Robert C Grumet, Scott Hadley, Matthew V Diltz, Thay Q Lee, Ranjan Gupta

**Affiliations:** ^1^Department of Orthopaedic Surgery, University of CaliforniaIrvineUSA; ^2^Orthopedic Biomechanics Laboratory, VA Long Beach Healthcare System>Long Beach, CAUSA

## Abstract

**Background and purpose** The New Zealand white rabbit subscapularis tendon passes under a bony arch to insert on the lesser tubercle of the humerus in a manner analogous to the supraspinatus tendon in humans. We assessed whether this unique anatomy may provide a new animal model of the shoulder to improve our understanding of rotator cuff pathology.

**Methods** The dimensions of the rotator cuff insertions (subscapularis, supraspinatus, and infraspinatus) were measured on 10 fresh frozen cadaveric New Zealand white rabbit shoulders. Mechanical testing was performed on 8 fresh frozen subscapularis insertions (4 matched pairs). Video analysis of the gait cycle was performed on 2 live animals.

**Results** The origins, insertions, and innervations of the rabbit rotator cuff musculature are analogous to those in humans. However, the rabbit acromion is a rudimentary structure with only the infraspinatus and teres minor muscles passing beneath. Furthermore, at the point where the infraspinatus passes under the arch, it is muscular rather than tendinous. The anterior aspect of the glenohumeral joint contains an additional bony tunnel with its boundaries being the tuberculum supraglenoidale laterally, the coracoideus process superiorly, the tuberculum infraglenoidale inferiorly, and the coracobrachialis muscle medially. The origin of the rabbit subscapularis muscle resides on the anterior scapula. The subscapularis tendon then traverses this bony tunnel prior to its insertion on the lesser tubercle of the humerus. Video analysis and anatomic dissections confirmed excursion of the subscapularis tendon within this bony tunnel throughout the gait cycle. The subscapularis footprint on the proximal humerus measured 6.8 mm (SD 0.29) × 2.5 mm (SD 0.17). Mechanical testing of the subscapularis tendon showed the stiffness to range from 57 to 117 N/mm (SD 23). Ultimate yield ranged from 88 to 215 N (SD 518). The elastic modulus of the rabbit tendon was 56 MPa. 6 of the 8 subscapularis tendons failed at the tendon mid-substance; the other 2 failed at the bony insertion.

**Interpretation** The unique anatomic architecture and the mechanical characteristics of the rabbit subscapularis muscle provide an opportunity to improve our understanding of rotator cuff pathology.

## Introduction

There is still a great deal to be discovered about rotator cuff tendinopathy, its pathogenesis, and post-surgical healing. In an effort to increase this understanding, many animal shoulder models have been used—including the rat, rabbit, goat, and sheep (Gerber et al. 2004). Perhaps the most intensively studied animal model for rotator cuff disease is the rat. Soslowski et al. (2000), Carpenter et al. 1998a, b, and others (Schneeberger et al. 1998, Gimbel et al. 2004, Perry et al. 2005) have defined the rat shoulder as an appropriate animal model of the human condition. Specifically, the anatomic relationship of the supraspinatus tendon to the acromion in the rat shoulder is analogous to that in humans. Using this model, the roles of overuse as well as intrinsic and extrinsic factors in rotator cuff tendinopathy have begun to be delineated (Schneeberger et al. 1998, Soslowski et al. 2000). In addition, several investigators (Barton et al. 2005, Perry et al. 2005) have studied the histological and molecular changes associated with rotator cuff tears in the rat model. Limitations to this model, however, include the fact that the shoulder in a quadruped animal is a weight-bearing joint, unlike in humans. The rat shoulder is small in size, making surgical procedures tedious and tissues delicate to handle. Additionally, Schneeberger et al. (1998) recognized that the portion of the rat supraspinatus muscle that passes under the acromial arch is muscular, and not tendinous as it is in the human. Finally, Barton et al. (2005) recognized a lack of fat accumulation in the surgically created rotator cuff tear in rats, which again contrasts with the human condition (Gladstone et al. 2007).

There has, however, been rather limited use of the rabbit shoulder to investigate the pathogenesis of rotator cuff pathology (Bjorkenheim 1989, Fabis et al. 2001, Choi et al. 2002, Uthoff et al. 2003). In the development of a reliable animal model for research, several criteria must be met (Soslowski et al. 1996): there must be a precise understanding of the anatomical relationship of the structures to be studied, one must be able to simulate the desired pathological condition and record the desired outcome measures, and finally, the model must be applicable to the human condition. Our goal was to assess whether the rabbit shoulder would meet these criteria. In addition, this model would give us the opportunity to use a larger animal with more substantial tissue mass than in the rat model.

## Material and methods

All procedures and protocols were approved by the Institutional Animal Care and Use Committee at the VA Long Beach Healthcare System. The animal research facility is fully accredited by Association for the Assessment and Accreditation of Laboratory Animal Care. All rabbits were male juveniles weighing between 3.4 and 3.8 kg.

### Anatomy (Figure 1)

Bilateral shoulders of 5 fresh frozen cadaveric male New Zealand white rabbits were dissected to define the anatomy of the rotator cuff, bony scapula, glenohumeral joint, brachial plexus, and rotator cuff musculature innervation. Each shoulder was disarticulated at the level of the scapulothoracic articulation. The skin and subcutaneous tissues were removed. The origin and insertion of each rotator cuff muscle was identified on the scapula and proximal humerus. The rotator cuff insertions on the proximal humerus were released to expose the underlying glenohumeral capsule. The capsule was subsequently incised and the glenohumeral joint disarticulated. With the glenoid surface exposed, the origin of each rotator cuff muscle was dissected free from the bony attachment on the scapula. The scapular anatomy was analyzed further and the unique architecture characterized (Winegard 1985).

**Figure 1.**
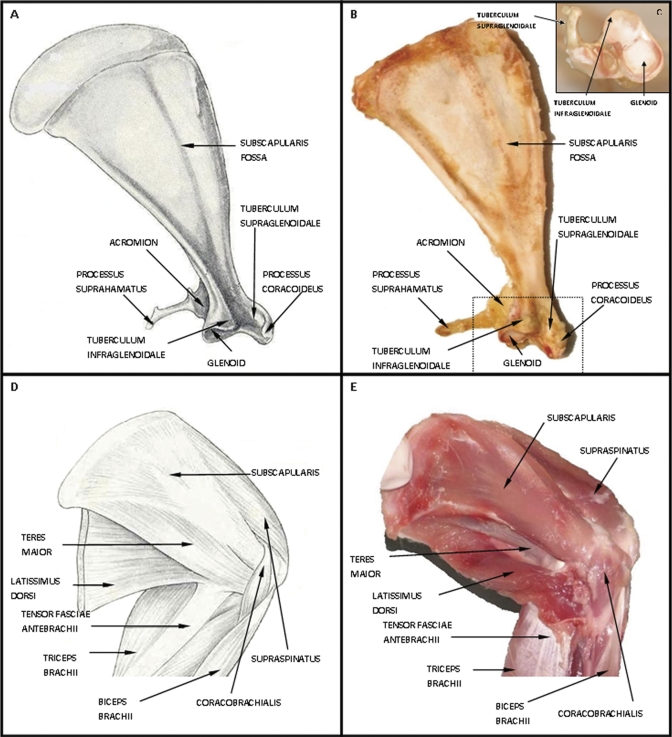
A and B. Schematic drawing and image representing anterior rabbit scapula with musculature and humerus removed demonstrating the bony tunnel of the subscapularis tendon. C. Demonstrates the rabbit scapula on end with an additional view of the bony tunnel. D and E. Schematic drawing and image representing the anterior rabbit scapula with the attached musculature.

Digital calipers (accuracy 0.02, resolution 0.01, precision 0.005 mm; Mitutoyo Corp., Japan) were used to measure the mediolateral and superoinferior dimension of these 10 rabbit supraspinatus, infraspinatus, and subscapularis insertions to the nearest 0.01 mm (5 matched pairs). Each measurement was taken 3 times and measurements were recorded by a single investigator (MD).

### Function

**Figure 2.**
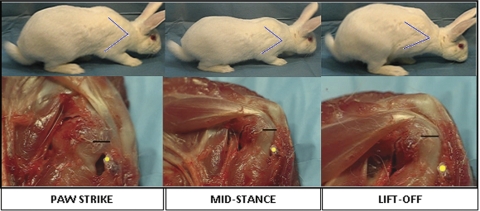
The top images represent the 3 phases of the right forelimb gait cycle in the rabbit, from paw strike to lift-off. Using video analysis, the scapulohumeral angle was calculated at each phase of the gait cycle. These angles were then translated to the dissected specimen (represented by the blue line on the rabbit forelimb). The lower series of images relates the translated scapulohumeral angle to the dissected specimen. The yellow dot represents the coracoid process and the black line the subscapularis tendon insertion on the humerus. Gross inspection reveals that the tendon has excursion within the tunnel from paw strike to lift-off. This is represented by the increase in distance from the yellow dot (corocoid process) to the black line (subscapularis insertion).

A video recording was taken of 2 male rabbits during forward locomotion to analyze their gait cycle (paw strike, mid-stance, and lift-off). Video imaging analysis using WINanalyze software (WINanalyze 2D, version 2.03) was used to digitally mark the relative position of the humeral shaft and scapular spine throughout the gait cycle. The scapulohumeral angle was then extrapolated at each phase of the gait cycle—paw strike, mid-stance, and lift-off—using the video software. These measurements were then recorded and translated to the gross rabbit shoulder specimens. The anterior portion of the rabbit scapula and proximal humerus was dissected free from skin and subcutaneous tissues. The musculature and glenohumeral articulation were kept intact. A yellow paint mark was placed on the coracoideus process of the scapula, the most superior position of the static bony tunnel. This anatomic structure can be easily visualized from the anterior portion of the shoulder. An additional black paint mark was placed on the insertion of the subscapularis tendon on the proximal humerus (Figure 2). The dissected cadaveric shoulder was then placed at each phase of the gait cycle using a goniometer, from paw strike to lift-off, using the previously defined scapulohumeral angle from the video analysis. The relative position of these two marks was inspected visually throughout the cycle.

### Mechanical testing of the subscapularis tendon-bone complex

**Figure 3.**
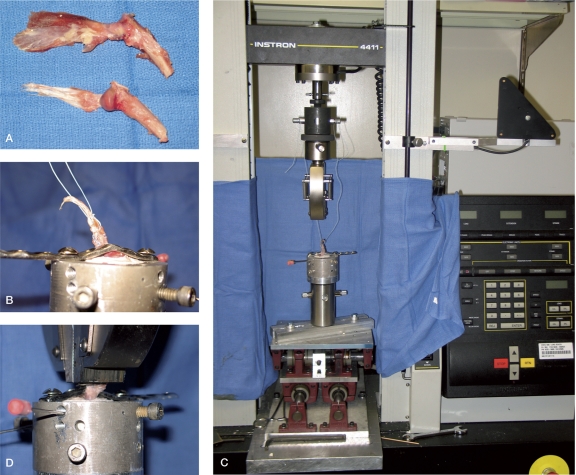
A. Anterior/posterior view subscapularis tendon insertion on proximal humerus. B. Humerus potted in polyvinylchloride pipe, substance of tendon sutured and secured with small frag plate. C. Instron machine. D. Humerus in position with tendon in clamp of Instron machine.

The rabbit subscapularis tendon-bone complex was tested using a custom fixture clamping system, an Instron materials testing machine (Instron Corp., Canton, MA), and a video digitizing system (WINanalyze software). 8 shoulders (4 matched pairs) of fresh frozen cadaveric rabbits were inspected for congenital shoulder anomalies, advanced arthritis, or rotator cuff injury. The subscapularis muscle was then dissected from its scapular origin and the tendon insertion preserved on the proximal humerus. The specimen was then dissected free from all other muscular, capsular, and ligamentous constraints, leaving the rabbit subscapularis tendon-bone complex (Figure 3). The humerus was then sharply transected at the midshaft level. Specimens were kept moist with normal saline solution during all phases of dissection, preparation, and testing. The humerus was then potted using plaster of Paris in a rigid polyvinylchloride pipe and secured using 2 K-wires. The humerus was potted at an angle equivalent such that the direction of pull of the tendon would be directly superior and along the axes of the subscapularis tendon fibers (Figure 3). In an effort to minimize soft tissue slippage in the clamp, suture (Ethicon Inc., Cincinnati, OH) was placed into the substance of the suscapularis tendon, taking care to preserve the tendon-bone interface.

Each specimen was loaded into the Instron and double-sided sand paper was placed between the jaws of the clamp and the tendon. A large fragment screw was used to secure the humeral head to the shaft and to prevent failure at the humeral physis with loading, taking care not to violate the bone-tendon interface. In addition, the subscapularis tendon was trimmed both superiorly and inferiorly leaving a 4-mm tendon insertion on the proximal humerus. This width was measured using the digital calipers. For deformation analysis, digital markers were placed at the level of the bony insertion of the tendon and the insertion of the tendon into the clamp. Video analysis software was then used to track the change in tendon length during load-to-failure analysis. Finally, for tensile testing of the rabbit subscapularis tendon-bone complex, a preload of 20 N was first applied to the specimen for 30 seconds. The tendon was then loaded to failure at a rate of 10 mm/min. The failure was recorded with a high-resolution digital video camera and later inserted into the WINanalyze software program for deformation analysis and cross-sectional area measurements. Specifically, the cross-sectional area of the tendon was calculated at the tendon mid-substance nearest to the site of failure. The strain was calculated using WINanalyze digital markers placed on the tendon on either side of the failure site. The structural properties of the subscapularis tendon-bone complex and the material properties of the subscapularis tendon were then calculated. This included linear stiffness, elastic modulus, yield and ultimate load, and stress. The mechanical properties of different failure modes were compared using the Student t test and the level of significance was set at p < 0.05.

## Results

### Anatomy

Analogous to the human condition, the rabbit rotator cuff is formed by the supraspinatus, infraspinatus, teres minor, and subscapularis tendons (Table). The rabbit subscapularis originates from the subscapularis fossa on the anterior aspect of the scapula. The tendon crosses the glenohumeral joint to insert on the lesser tubercle of the anterior proximal humerus (Figure 1).

**Table:** 

Muscle	Origin	Insertion	Innervation
Supraspinatus	Supraspinatus fossa of scapula	Greater tubercle of humerus	Suprascapular nerve
Infraspinatus	Infraspinatus fossa of scapula	Greater tubercle of humerus	Suprascapular nerve
Teres minor	Ventral portion of axillary border of scapula	Greater tubercle of humerus	Axillary nerve
Subscapularis	Anterior face of scapula	Lesser tubercle of humerus	Subscapular nerve

With regard to the bony anatomy of the rabbit scapula, the acromion is a relatively rudimentary structure (Figure 1). The infraspinatus and teres minor muscles pass under this acromial arch to insert on the greater tubercle, whereas the supraspinatus does not. Furthermore, the infraspinatus is muscular, rather than tendinous, at the junction under the acromion. The most unique attribute of the rabbit scapula is an additional bony prominence, or tunnel, on the anterior aspect of the glenohumeral joint (Figure 1). This bony tunnel is 3-sided, with the lateral wall of this structure consisting of the tuberculum supraglenoidale; the roof is the process coracoideus, and finally the floor is the tuberculum infraglenoidale. The tendon of the subscapularis muscle travels through this bony tunnel prior to its insertion on the lesser tubercle of the humerus. Anterior to the tunnel and tendon is the coracobrachialis muscle, the “medial” wall of the tunnel.

The mean subscapularis superoinferior dimension (length) was 6.8 mm (SD 0.30) and the mean mediolateral dimension (width) was 2.5 mm (SD 0.17).

### Subscapularis function

Gross examination revealed subscapularis functions in the rabbit that were similar to those of the human subscapularis. Combined with the additional rotator cuff muscles around the proximal humerus, it aids in compression of the shallow glenohumeral joint. In addition, the subscapularis assists in forward elevation of the forelimb and internal rotation.

The scapulohumeral angle of the rabbit during forward locomotion at each phase of the gait cycle was extrapolated to the dissected specimen. The specimen was then placed at the corresponding scapulohumeral angle as measured by a goniometer. A yellow mark was placed on the processus coracoideus and a line was placed on the subscapularis insertion to the lesser tubercle of the proximal humerus. On gross inspection, as the glenohumeral joint was moved from maximal extension (paw strike) to maximal flexion (lift-off), the distance of the subscapularis insertion from the processus coracoideus increased. During forward locomotion, the forelimb was positioned such that excursion of the subscapularis tendon occurs within the bony tunnel as described above (Figure 2).

### Mechanical testing of the subscapularis tendon – bone complex

**Figure 4.**
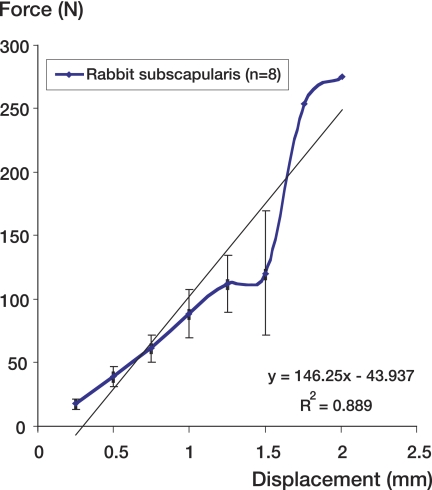
Force (N) versus displacement (mm) curve of rabbit subscapularis tendon. The average ultimate load was 112 N (SEM 23). The averaged ultimate deformation was 1.3 mm (SEM 0.15) and the stiffness (slope) was therefore 146 N/mm

6 of the 8 specimens failed at the tendon mid-substance; 2 specimens failed at the bone-tendon insertion. The two failures at the bone-tendon interface were in different animals. The ultimate load of the subscapularis insertion varied from 15 N to 275 N. The average ultimate load was 112 N (SEM 23). The averaged ultimate deformation was 1.3 mm (SEM 0.15) and the stiffness was therefore 146 N/mm (Figure 4).

**Figure 5.**
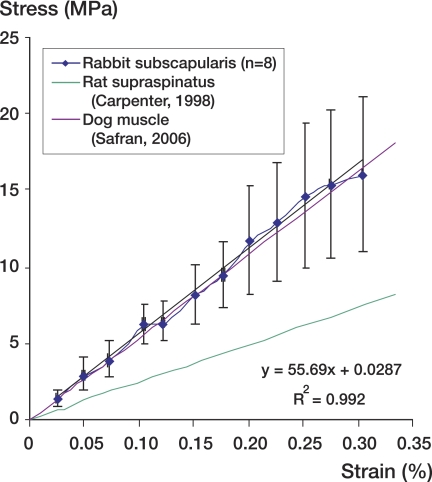
Stress (MPa) versus strain (%) comparing elastic modulus of rabbit subscapularis tendon to previous animal models for rotator cuff pathology. The rabbit subscapularis tendon has an elastic modulus of 56 MPa (R^2^=0.99). This modulus most closely correlates with the dog model for rotator cuff pathology 55 MPa.

The stress-strain curve demonstrated an average stress at failure of 8.2 MPa (SEM 3.1) and an average strain at failure of 0.16% (SEM 0.04). The linear modulus calculated from the stress-strain curve was 56 MPa (Figure 5).

With respect to ultimate load, there was no statistically significant difference (p = 0.4) between the failures of tendon at mid-substance and at the bony insertion: 105 N (SEM 34) and 133 N (SEM 66), respectively. Similarly, there was no significant difference (p = 0.3) between these two groups with respect to ultimate deformation. Ultimate deformations at mid-substance and at the bony insertion were 1.3 mm (SEM 0.24) and 1.48 mm (SEM 0.05).

Finally, no statistically significant difference (p = 0.5) was found between the stress at mid-substance failure 8.1 MPa (SEM 3.3) and at bony insertion failure 8.5 MPa (SEM 4.6).

## Discussion

There has been limited application of the rabbit shoulder to the study of rotator cuff disease, and it has been used mainly to evaluate the histological and mechanical changes that result from detachment of the rabbit supraspinatus tendon (Gupta and Lee 2007).

We have found the rabbit subscapularis muscle to be an accurate and reliable animal model for the study of rotator cuff tendinopathy, given its unique anatomical, histological, and mechanical properties. We identified the unique relationship of the rabbit subscapularis tendon and scapular bony tunnel (Figure 1). This condition is similar to what is found in the human rotator cuff and the supraspinatus traveling under the bony acromion prior to its insertion on the greater tubercle of the humerus. The role of a bony tunnel, and thus extrinsic compression, in the development of rotator cuff pathology is unclear (Soslowski et al. 2002). To prove or disprove the theory of extrinsic factors in the role of rotator cuff pathology in an animal model, it must mimic the human condition (Neer 1983). To our knowledge, the rabbit subscapularis tendon traveling in its bony tunnel is the only model that is analogous to the human anatomy. The widely accepted shoulder model of the rat has analogous anatomy to that of the human, with a supraspinatus traveling under the acromion. However, the portion of the rat supraspinatus muscle that passes under the acromial arch is muscular—not tendinous, as it is in humans (Schneeberger et al. 1998, Gerber et al. 1999). Barton et al. (2005) recognized a lack of fat accumulation in the surgically created rotator cuff tear in rats, which again is contradictory to the human condition (Gladstone et al. 2007).

In addition, our video analyses confirmed the excursion of the tendon within the tunnel during locomotion (Figure 2). To evaluate the role of extrinsic factors (bony tunnel), it is important to be absolutely sure that the tendon moves within the tunnel during normal forward locomotion. This motion is similar the supraspinatus tendon moving under the acromion during elevation and abduction of the humerus in humans.

The footprint of the human has been well elucidated (Richards et al. 2003, Ruotolo et al. 2004). The anteroposterior dimension of the human supraspinatus insertion is 25 mm (SD 2.4). The mean superior to inferior tendon thickness at the rotator interval is 12 mm, 12 mm at mid-tendon, and 10–12 mm at the posterior edge. The corresponding subscapularis in the rabbit has a mean width of 6.8 mm (SD 0.30). Thus, the approximate ratio of the human supraspinatus footprint to the rabbit subscapularis tendon footprint is approximately 4:1. We believe that the similarity of these dimensions is important for future studies of rotator cuff repair techniques.

Finally, the rotator cuff tendon-bone insertion is an important consideration in defining our model. We examined the pullout strength of the subscapularis tendon insertion on the proximal humerus. Despite consistencies in harvesting, rabbit size, age, sex, processing and pullout technique, the structural properties of the subscapularis had a wide range, however, including ultimate yield and ultimate deformation. This may be due in part to the length of time the rabbits were frozen, the number of times the rabbit shoulders were thawed, and the temperature of the tendon at the time of failure. Woo et al. (1986) evaluated the effects of prolonged postmortem frozen storage on the structural properties of the rabbit medial collateral ligament (MCL). They concluded that careful control of the freezing had no effect on the properties of the MCL. Our rabbit subscapularis tendon was frozen at –20ºC at an average of 2 weeks before mechanical testing. Prior to testing, the rabbits were thawed and dissected to expose the proximal humerus and subscapularis insertion. These dissected specimens were then frozen again and stored. Before final mechanical testing, the specimens were thawed for a second time, potted, and loaded in the Instron machine. We suspect that this thawing and freezing of the tendon twice may have had an effect on the structural properties. In addition, the temperature at the time of failure may have an influence on the structural properties of the tendon. The time between thawing and testing was not specifically controlled and may therefore have influenced the data. It is also known that when performing mechanical testing of a tissue specimen, variations in the structural properties can be expected due to differences in dimensions of the tendon, temperature, and so on. Finally, McMahon et al. (2001) have defined differences in the non-recoverable deformation along the length of a tissue specimen during tensile testing. The amount of variation varies depending on the location of the failure.

Despite the differences in the structural properties of the tendon, the material properties of the rabbit subscapularis were relatively consistent and representative of the mechanical properties seen by other animal investigators (Figure 5). Specifically, the rabbit subscapularis has an elastic modulus of 56 MPa (R2 = 0.99). This modulus corresponds most closely with the dog model for rotator cuff pathology (55MPa) (Safran et al. 2005).

Many animal models have been created to describe the pathogenesis and post-surgical healing of human rotator cuff pathologies (Bjorkenheim 1989, Gerber et al. 1999, Soslowski 2000, Safran et al. 2005). The anatomy of the rabbit subscapularis muscle most closely resembles the human situation, in terms of the relationship of the rotator cuff tendon with the anterior bony process. The rabbit is larger than the more widely used rat, which affords an opportunity for more precise and consistent surgical procedures. Also, significant work has been done to define the histological and molecular changes in the rabbit after supraspinatus tear (Choi et al. 2002, Perry et al. 2005), thus laying the groundwork for interpretation of a subscapularis tendon injury. However, the rabbit is a quadraped with a weight-bearing glenohumeral joint. This characteristic may change the histological and mechanical response of the shoulder to injury, given a larger joint reactive force experienced with weight bearing. Additionally, as mentioned previously there are inconsistencies in the mechanical properties of the tendon. Further testing, with a larger sample size, will probably be necessary in the future when preparing to compare the pullout strength of intact and repaired tendon.

In future, our work will be to continue validation of this model by evaluating the histological and molecular changes seen after tendon transection, and development of a chronic injury model. In particular, previous investigators have defined muscular atrophy, increased cellularity/vascularity, and increased fatty degeneration after tendon injury (Bjorkenheim et al. 1989, Carpenter et al. 1998a, b, Soslowski et al. 2000, Barton et al. 2005). The long-term goals include beginning to understand the possibility of neuromuscular unit injury being a potential cause of irreversible tendon and muscle changes after a rotator cuff tear.
